# Nanoscale insights into doping behavior, particle size and surface effects in trivalent metal doped SnO_2_

**DOI:** 10.1038/s41598-017-09026-2

**Published:** 2017-08-29

**Authors:** Bogdan Cojocaru, Daniel Avram, Vadim Kessler, Vasile Parvulescu, Gulaim Seisenbaeva, Carmen Tiseanu

**Affiliations:** 1 Department of Organic Chemistry, Biochemistry and Catalysis, University of Bucharest, 4-12 Regina Elisabeta Bvd, Bucharest, 030016 Romania; 2 National Institute for Laser, Plasma and Radiation Physics, P.O. Box MG-36, RO 76900 Bucharest-Magurele, Romania; 3 Department of Molecular Sciences, Biocenter, SLU, Box 7015, SE-75007 Uppsala, Sweden

## Abstract

Despite considerable research, the location of an aliovalent dopant into SnO_2_ nanoparticles is far to be clarified. The aim of the present study on trivalent lanthanide doped SnO_2_ is to differentiate between substitutional *versus* interstitial and surface *versus* bulk doping, delineate the bulk and surface defects induced by doping and establish an *intrinsic* dopant distribution. We evidence for the first time a complex distribution of intrinsic nature composed of substitutional isolated, substitutional associates with defects as well as surface centers. Such multi-modal distribution is revealed for Eu and Sm, while Pr, Tb and Dy appear to be distributed mostly on the SnO_2_ surface. Like the previously reported case of Eu, Sm displays a long-lived luminescence decaying in the hundreds of ms scale which is likely related to a selective interaction between the traps and the substitutional isolated center. Analyzing the time-gated luminescence, we conclude that the local lattice environment of the lattice Sn is not affected by the particle size, being remarkably similar in the ~2 and 20 nm particles. The photocatalytic measurements employed as a probe tool confirm the conclusions from the luminescence measurements concerning the nature of defects and the temperature induced migration of lanthanide dopants.

## Introduction

There has been considerable research over the past decades on the n-type wide band gap metal oxide semiconductor, tin oxide (SnO_2_) due to its broad spectrum of applications. It is commonly used in transparent conducting electrodes and chemical sensors^1, 2^ production of batteries in conjunction with carbon based materials^3^, photocatalysts either in pure state, doped with non-lanthanide^4^, lanthanide ions (Ln)^5^ or in combination with another oxide (for example SnO_2_/TiO_2_^6^, or SnO_2_/ZnO^7^) as well as photocatalysts with a post-illumination photocatalytic “memory”^8^. SnO_2_ has the rutile-type tetragonal structure belonging to the P_42_/*mnm* space group (lattice parameters a = b = 4.738 Å and c = 3.187 Å) with a band energy-gap situated between 3.5 and 3.8 eV according to both experimental results and theoretical calculations^9, 10^. Band- gap engineering has been used as an effective way to tune the band structure and optoelectronic properties of this oxide^11^. For this purpose, SnO_2_ has been synthetized by exploiting numerous approaches such as precipitation^12^, photochemical growth at the air–water interface^9, 10^, thermal decomposition^13^, sol-gel^14^, surfactant-assisted solvothermal^10^, hydrothermal synthesis^15, 16^ and sono-chemical method^17^.

Doping of SnO_2_ nanomaterials with metal cations proved to be a successful tool for tailoring their electrical, optical, and microstructural properties. The luminescence of pure SnO_2_, observed in the UV and/or visible region (350–550 nm) is generally correlated with the presence of crystalline defects resulting from the various synthesis processes^18, 19^. The literature agrees towards the oxygen vacancies as the most probable candidates for the recombination centers in the emission processes of SnO_2_^18, 19^. Of the various metal dopants of SnO_2_, the aliovalent Ce^3+^ ^18, 19^, Mn^2+^ ^18, 19^ Co^2+^ ^20^, Ni^2+^ ^21^ or Cr^3+^ ^22^ revealed significant information on the relationships between doping, defects related luminescence, surface effects, changes in morphology and particle size.

Selection of a luminescence approach that is sensitive more to the local structure is expected to expose better the dopant location and distribution in SnO_2_. The trivalent Eu Ln metal with its characteristic orange/red emission is known to be highly sensitive to its local environment^23^. This explains why the Eu represents the most frequently investigated luminescent dopant for SnO_2_ with first reports dating back four decades ago^24, 25^. The information extracted from Eu luminescence can reveal whether the dopants segregate on the surface of nanosized SnO_2_ or/and they enter the oxide lattice *via* interstitial or substitutional way. In case of substitutional doping, substitution of Sn(4+) by bulkier Eu(3+) leads to both strain and electric effects due to the mismatch of both ionic radii (0.076 nm compared to 0.095 nm, in six-fold coordination^26^) and valence. The oxygen vacancies arising from charge-compensation can, in principle, locate either in the close vicinity or distant to Eu dopants leading to Eu- defects associates or substitutional isolated center, respectively. There are several reports that describe the emission shape of Eu as being dominated by relatively strong, three magnetic dipole (MD) emission lines around 590 nm, corresponding to ^5^D_0_–^7^F_1_ transition, in good agreement with the selection rules for the inversion low symmetry (C_2h_) at Sn sites. It is also established that SnO_2_ host acts as an efficient antenna that absorbs the UV excitation and subsequently transfers the excitation energy to the substitutional isolated Eu, compensating thus for the intrinsic low absorptions of the parity forbidden f – f transitions^27, 28^. Recently, the co-existence of uniform and exponential distributions in trap depths along with the thermal activation mechanism were suggested to explain a unique afterglow decay behavior in Eu - SnO_2_^27^. It is established that, as the particle size decreases, the increasing number of surface and interface atoms generates stress/strain and concomitant structural perturbations^28^. By use of luminescence spectroscopy, a strongly distorted Eu environment has been suggested to occur in SnO_2_ nanoparticles with dimension close to the exciton Bohr radius (around 2 nm) compared to greater sized nanoparticles^29^. In addition, our literature survey evidences that a complex Eu related emission with both broad and narrow spectral features accompanies the characteristic emission of substitutional isolated Eu centers. The shape of this emission varies strongly with Eu concentration, synthesis approaches, thermal treatment and excitation conditions used in luminescence measurements^28–34^. The origin of this emission is undecided in the literature, being tentatively related to either Eu – defect associates, impurity phases (such as Eu_2_O_3_ or Eu_2_Sn_2_O_7_) or some unknown phase. In all, the literature data evidence a complex emission of still unknown origin for the Eu-SnO_2_ system. Elucidation of the specific location of the aliovalent dopant into the lattice is crucial since this influence significantly the functional properties of doped SnO_2_ nanomaterials. As an example, for a different significant semiconductor, anatase TiO_2_, it has been established that only Ln that enter substitutional in the TiO_2_ lattice contribute to the change of electronic structure and light absorption efficiency of the host^35^. For this host, substitutional doping mechanism was advanced at least for Nd, Sm, Eu, Er lanthanide ions^36^, whilst for Eu and Sm several lattice sites were identified already by site selective luminescence spectroscopy^36–38^.

To date, besides Eu, only Er Ln has been demonstrated by luminescence studies to substitute for the Sn lattice sites in the inversion C_2h_ sites as an isolated substitutional dopant^39^. Indeed, Chen *et al*.^38^ measured an ultranarrow long-lived emission around 1500 nm which corresponds to the ^4^I_13/2_–^4^I_15/2_ transition having a strong magnetic dipole component^40^. For Nd, substitutional doping was suggested by luminescence and near edge X-ray absorption fine structure (NEXAFS) measurements^41^; however, the observance of a relatively strong ^4^F_3/2_ emission of Nd in the range of 800 to 1400 nm which is predominantly of electric dipolar nature sustains more a substitutional doping in association with a nearby defect. In such case, the association of the lanthanide with a defect in the nearest neighbour position remove the inversion symmetry around the dopant and thus the electric dipole transitions become allowed and their intensity dominate the spectra. For Sm^42, 43^, Tb^44^ or Dy^45, 46^ doped SnO_2_, the reported emission shapes deviate from the ones expected for an inversion local symmetry^47–49^, and thus, the isolated substitutional doping route remain uncertain.

It can be thus concluded that a critical question to be answered when studying doped SnO_2_ nanoparticles is *How do we know if SnO*_2_ *nanoparticles have been successfully doped?* In this context, our study on the trivalent Ln doped SnO_2_ is aiming to differentiate between substitutional *versus* interstitial and surface *versus* bulk doping, delineate the bulk and surface defects induced by doping and establish an intrinsic dopant distribution. The experimental confirmation of successful doping of semiconductor nanoparticles with luminescent lanthanide activators is usually done in literature by a combination of the *ex situ* luminescence, X-ray diffraction (XRD), Diffuse Reflectance in the Ultraviolet/Visible (DR-UV/Vis) Raman, and Transmission Electron (TEM) or Scanning Electron (SEM) microscopy methods and techniques. Since for SnO_2_, reports show that the distribution of various metal dopants changes dramatically with temperature, from ordered, lattice sites to disordered, likely surface regions^50^, we use the *in situ* XRD, Raman and luminescence to provide additional insight into the dynamic evolution of dopant environment during thermal treatment^51^. In addition to conventional bulk doping by rapid hydrothermal or sol-gel methods, the impregnation of Ln on pre-calcined SnO_2_ nanoparticles was tested to differentiate between the bulk and surface defects. Photocatalytic oxidation of phenol was used as a reaction tool to further differentiate between the natures of Ln induced defects.

## Results and Discussion

### Overview of the texture, morphology and structure properties

As synthetized pure SnO_2_ consists of well-defined 2–4 nm sized nanoparticles with a near-spherical shape (Figure S1) aggregated into rather uniform structure with wormhole mesoporosity, originating from the diffusion controlled hydrolysis-polycondensation mechanism^52^. With thermal treatment at 400 °C, more strongly intergrown structure of 5–6 nm particles are evidenced along with sharp decrease of the surface area and increase of the pore size which continues with the increase of temperature at 700 and 1000 °C (Table 1). Calcination at 700 °C led to a bimodal pore size distribution combining mesopores and macropores whilst further increase at 1000 °C induced formation of a compact nonporous oxide.

**Table 1:** BET surface area and pore size of SnO_2_ treated at different temperatures.

Sample	S_BET_ (m^2^/g)	Pore size (nm)
SnO_2_ as prepared	150	2.4
SnO_2_ 400 °C	47	4.6
SnO_2_ 700 °C	9.2	4.8; 29.6
SnO_2_ 1000 °C	0.3	—

**Figure 1:**
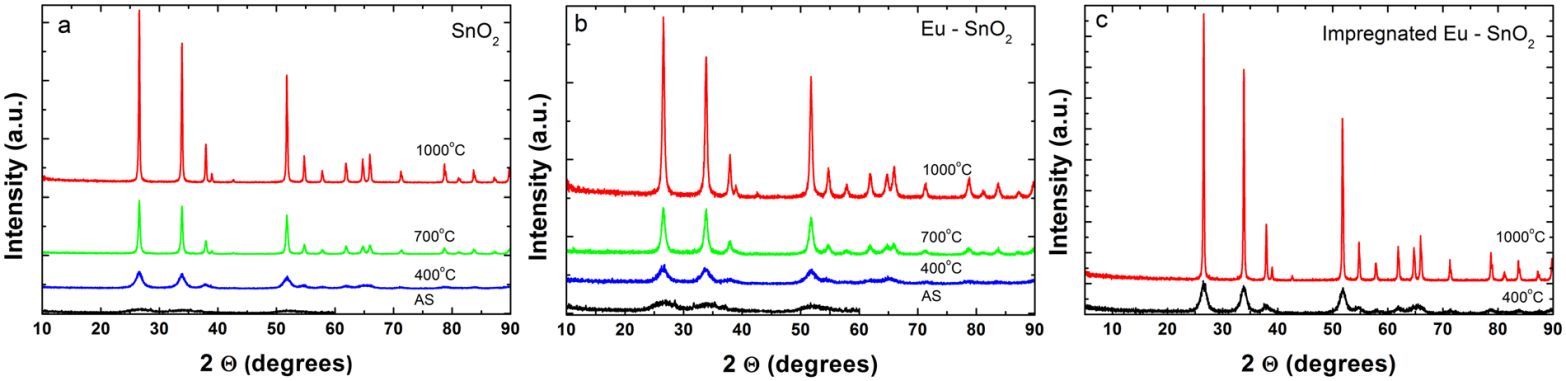
XRD patterns of SnO_2_ (**a**), Eu-SnO_2_ (**b**) and impregnated Eu-SnO_2_ (**c**, see text).

The purity of Ln (Eu, Sm) doped SnO_2_ samples was confirmed by X-ray fluorescence analysis (Figure S1). Doping SnO_2_ with 1% Ln did not cause any change in these textural properties and overall morphology. Generally, similar texture, morphology and structure properties were observed for Eu or Sm doped SnO_2_; therefore, we describe Eu- SnO_2_ in more detail. The *ex situ* XRD patterns of SnO_2_ and Eu-SnO_2_ calcined at 400, 700 and 1000 °C are presented in Fig. 1 (see also Figure S3 for the *in situ* with temperature XRD patterns of Eu-SnO_2_).

The diffraction angles at 26.3°, 33.8° and 51.8° from the diffractograms illustrated in Fig. 1, can be assigned to (110), (101) and (211) planes of the tetragonal SnO_2_ (PDF card 00-041-1445). In the limit of instrumental resolution, impurity phases, such as Eu_2_O_3_ or pyrochlore Eu_2_Sn_2_O_7_ were not detected. Broadening of XRD patterns with Eu doping together with the crystallite size and lattice parameters values listed in Table 2 demonstrate that Eu doping reduce the crystallite growth in large agreement with literature.

**Table 2:** Particle size, lattice constants and estimated band-gap of pure and Eu doped/impregnated SnO_2_.

Calcination temperature (°C)	Particle size (nm) ( ± 0.5 nm)			Lattice constants (Å)* (±0.05 Å)						Band-gap (eV)** (±0.05 eV)		
	SnO_2_	Eu-SnO_2_	Eu-SnO_2_ (I)	SnO_2_		Eu-SnO_2_		Eu-SnO_2_ (I)		SnO_2_	Eu-SnO_2_	Eu-SnO_2_ (I)
				a = b	c	a = b	c	a = b	c			
400	6	5	6	4.743	3.188	4.745	3.199	4.746	3.187	2.6	2.5	2.6
700	24	11	—	4.738	3.186	4.742	3.190	—	—	2.7	2.6	—
1000	40	19	44	4.741	3.190	4.745	3.191	4.738	3.186	2.9	2.7	3.2

*Lattice constants according to PDF 00-041-1445: a = b = 4.738 Å, c = 3.187 Å; Lattice constant from tetragonal SnO_2_ was calculated from the formula $\frac{1}{{d}^{2}}=\frac{{h}^{2}+{k}^{2}}{{a}^{2}}+\frac{{l}^{2}}{{c}^{2}}$, using (200) and (101) scattering planes from ~37.9 and 33.8 degrees, respectively. **Band gap values for uncalcined samples: 3.25 eV for SnO_2_, 3.23 eV for Eu-SnO_2_; (I) refers to impregnated samples (see text).

Similar effect was reported for other lanthanide ions and attributed to a solute drag and lattice distortion caused by the substitutional rather than surface doping^53, 54^. It is also worth noting that all crystallite sizes of the calcined samples exceed the quantum confinement region set by the exciton Bohr radius of SnO_2_ at 2.7 nm^55^.

**Figure 2:**
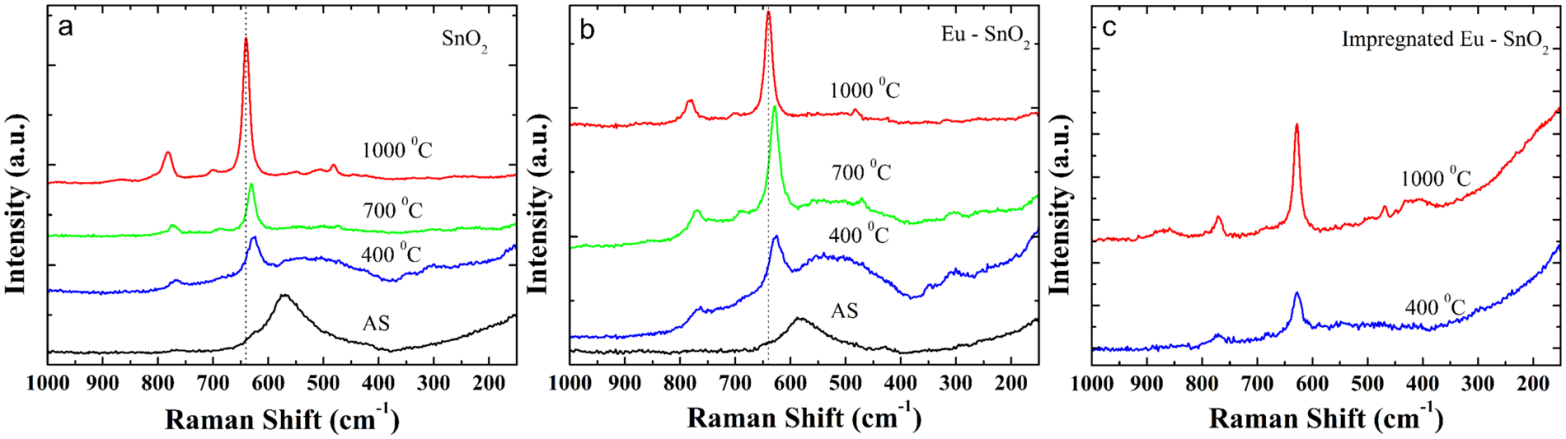
Raman spectra of SnO_2_ (**a**), Eu-SnO_2_ (**b**) and impregnated Eu-SnO_2_ (**c**, see text).

Figure 2 shows the Raman spectra of SnO_2_ and Eu-SnO_2_ samples. The as - synthetized pure sample presents a broad and intense band at 570 cm^−1^ which is typically attributed to surface effects along with weak band around 760 cm^−1^ corresponding to B_2g_ mode^56^. Samples calcined at 400, 700 and 1000 °C show in addition modes at 270–302, 474–510 (E_g_), 626–641(A_1g_) and 680–700 and 768–782 cm^−1^ (B_2g_) in good agreement with literature^57^. Along with the increase of the temperature, a shift of the A_1g_ and B_2g_ lines towards higher wavenumbers and of the E_g_ line to lower wavenumbers is observed, associated to the increase of the particle size^56^. It should be remarked that doping with Eu is leading both to a stronger intensity of the broad bands around 500–570 cm^−1^ and decrease of the particle size as shown by the XRD patterns (Fig. 1, Table 2) which confirm the surface nature of this phonon mode.

DRIFTs spectra of SnO_2_ and Eu-SnO_2_ exhibit the typical profile for SnO_2_^58^ (see Figure S4 and associated text). Pure samples show that the water content as well as hydroxyl absorption bands progressively disappear as the calcination temperature is increased whilst the presence of Eu appears to preserve more of the hydroxyl defects even at 1000 °C. The DR/UV-Vis diffuse reflectance spectra of SnO_2_ and Eu-SnO_2_ show a broad absorption band in the UV region (Figure S5). The shifts detected with the increase of the temperature may be attributed to the XRD observed changes in crystallinity and particle size (Fig. 1 and Table 2). The band-gaps (Eg) calculated by plotting (F(R)•hν)^2^ against hν^18^ (see also Experimental Section) are narrower than the value of 3.6 eV stated in literature^9, 10^ likely because of high concentration of oxygen vacancies/defects^59^. Further calcination at 1000 °C leads to the decrease of the band-gap up to 2.9 eV for pure SnO_2_ which narrowed to 2.7 eV for Eu-SnO_2_.

### *In situ* short to long range investigations in the mild calcination regime

**Figure 3:**
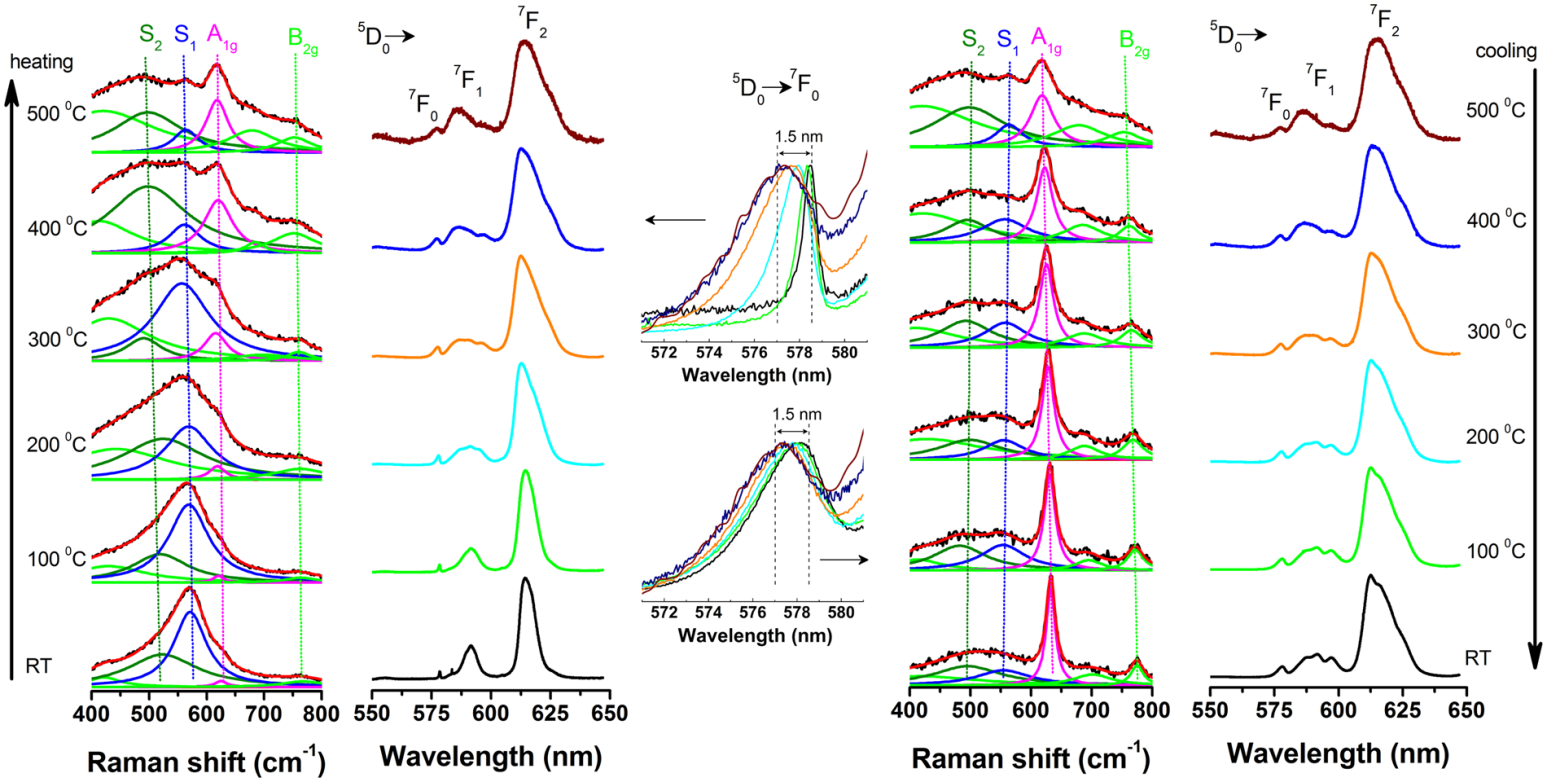
Evolution with temperature of the *in situ* Raman and *in situ* luminescence of Eu-SnO_2_ during heating (Left) and cooling cycles (Right). Middle inset detail the ^5^D_0_–^7^F_0_ (normalized) emission. Deconvolution of the Raman spectra was done by use of Lorentzian functions. Excitation wavelength used in Raman measurements is 514 nm.

To get deeper insight into how the particle size, surface features, defects and order/disorder effects influence *dynamically* the local structure around the trivalent dopant, we further analyzed the Raman, luminescence and XRD data obtained by *in sit*u measurements using Eu as a structural probe. *In situ* luminescence spectra were extracted from the *in situ* Raman spectra measured during heating and cooling cycles RT-500 °C-RT (RT stands for room-temperature, around 25 °C)^60^ while the i*n situ* XRD patterns of Eu-SnO_2_ (Figure S3) were measured during both heating and cooling cycles (RT- 1000 °C- RT). As shown in Fig. 3, the position, width and intensity of the Raman modes of Eu- SnO_2_ change continuously with temperature during heating cycle. Up to 250 °C the Raman spectra of Eu-SnO_2_ are dominated by a broad band around 570 cm^−1^ which relates to the small particle size/surface effects^56, 61^ or to amorphous nature of the particles^62^. The average particle size is estimated to increase slightly from 2–4 nm before calcination to 6 nm at 500 °C (Figure S3).

Besides this broad band (labelled as S_1_ in ref. 56) another broad phonon band is localized around 480 cm^−1^ (labelled as S_2_ in ref. 56). In the region of S_2_ band, the E_g_ mode around 470–480 cm^−1^ considered to be the most sensitive to oxygen vacancies, is expected to arise but its intensity is too low to be accounted for in the fitting^57^. Both S_1_ and S_2_ bands decrease in intensity with increase of particle size at the expense of A_1g_ mode at 620–626 cm^−1^. The two Raman modes were both assigned to surface effects, more specifically to surface layer (around 1 nm as width) formation of a nonstoichiometric SnO_2_ with different symmetries than SnO_2_. However, the bands seem to differentiate in what concerns their evolution with temperature: while before calcination their relative intensities are rather similar, at 500 °C the S_2_ band exceed in intensity by a factor of four that of S_1_ band, equaling again after cooling down to RT. During cooling cycle, a gradual shift towards red, from 620 to 633 cm^−1^ is measured for A_1g_ band parallels the slight increase of the particle size (Figure S3). As concern the *in situ* luminescence, this evidences broad shapes characteristic of surface Eu, dominated by strong emission around 612 nm which shifts and broadness continuously during heating and to less extent, during cooling cycles. To provide more insight into Eu distribution during the thermal treatment, the middle panel of Fig. 3 zoom the emission around ^5^D_0_–^7^F_0_ transition around 579 nm. This J = 0 − J = 0 transition is well known to be informative (in some conditions^23^) on the number of distinct Eu sites. Only single band assigned to average single Eu center could be detected with shape being progressively broadened and blue shifted from 578, 5 to 577, 4 nm during heating and then red shifted and slightly narrowed during cooling cycle. The luminescence evolution with temperature appears to be associated to either S_1_ or S_2_ or both phonon modes. The broadening of Eu emission during heating means an increased disorder around its nearest environment, likely induced by defects in the surface layer. Further series of experiments using pulsed laser excitation above 300 nm and long delay (time-gate mode) on the as-synthetized and calcined samples at 400 °C show a much weaker and narrower emission dominated by three ^5^D_0_–^7^F_1_ lines at 588 nm/593 nm/599 nm assigned to Eu substituting for Sn(4+) in C_2h_ inversion sites^24^. In all, the *in situ* Raman and *in situ* luminescence results confirm that in the absence of calcination most Eu reside on the surface of ca 2 nm sized SnO_2_ nanoparticles with only few Sn inner lattice sites being substituted by Eu. Mild calcination up to 500 °C lead to a significant reduction of surface effects while the redistribution of dopants along with generation of defects induced by weak incorporation process remain limited to the surface layer of ~2–6 nm sized particles.

### Substitutional doping, interaction with bulk defects and surface effects at high calcination temperatures

To date, literature generally describes a complex Eu related emission in SnO_2_ with both broad and narrow spectral features that changes strongly varies with Eu concentration, synthesis approaches, thermal treatment and excitation conditions used in luminescence measurements^28–32, 34, 63^. Here, we have extensively analyzed several tens of emission spectra of Eu-SnO_2_ calcined in air at 700 and 1000 °C at 80 and 300 K using various excitation wavelengths spanning the UV to Vis range and gate/time delays after the laser pulse (Figure S6). The specific aim of these investigations was to derive an intrinsic distribution of Eu described in terms of substitutional/interstitial doping, interaction with induced defects and surface effects.

**Figure 4:**
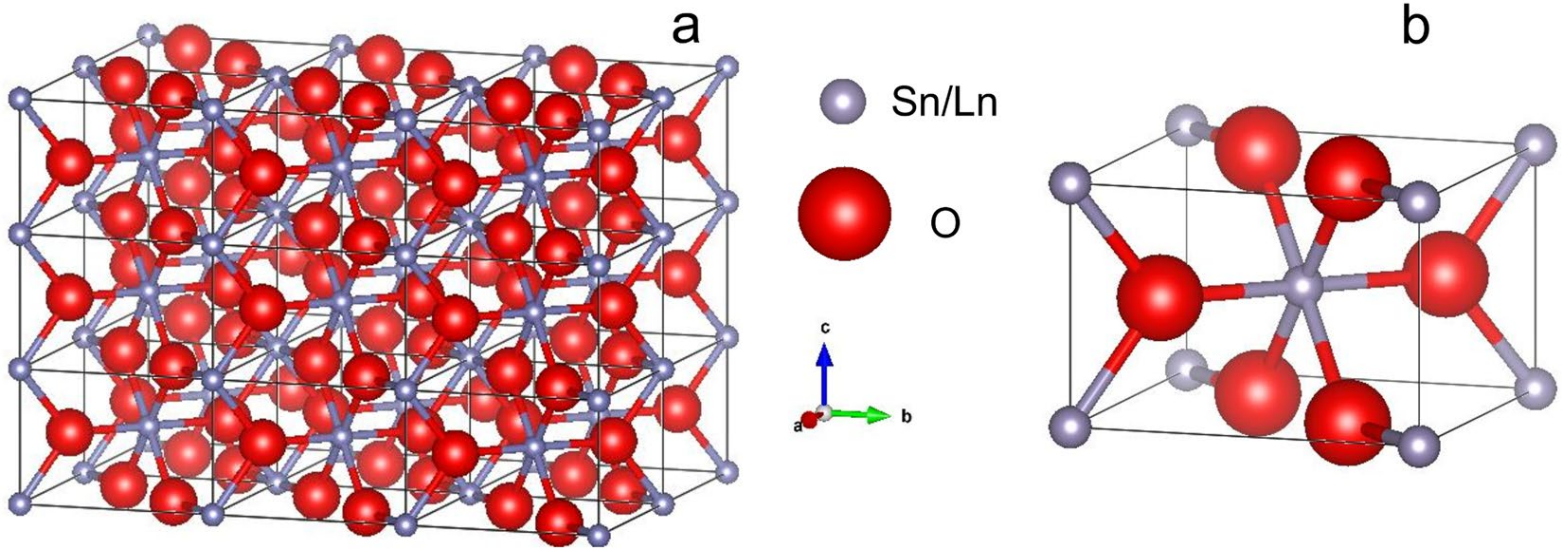
Crystal structure of SnO_2_ (**a**) Atomic arrangement in a rutile tetragonal unit cell with space group D_4h_^16^ (P_42/mnm_). A unit cell contains two tin ions and four oxygen ions; (**b**) Perfect SnO_6_ tetrahedron with C_2h_ local symmetry (with inversion center) around Sn.

**Figure 5:**
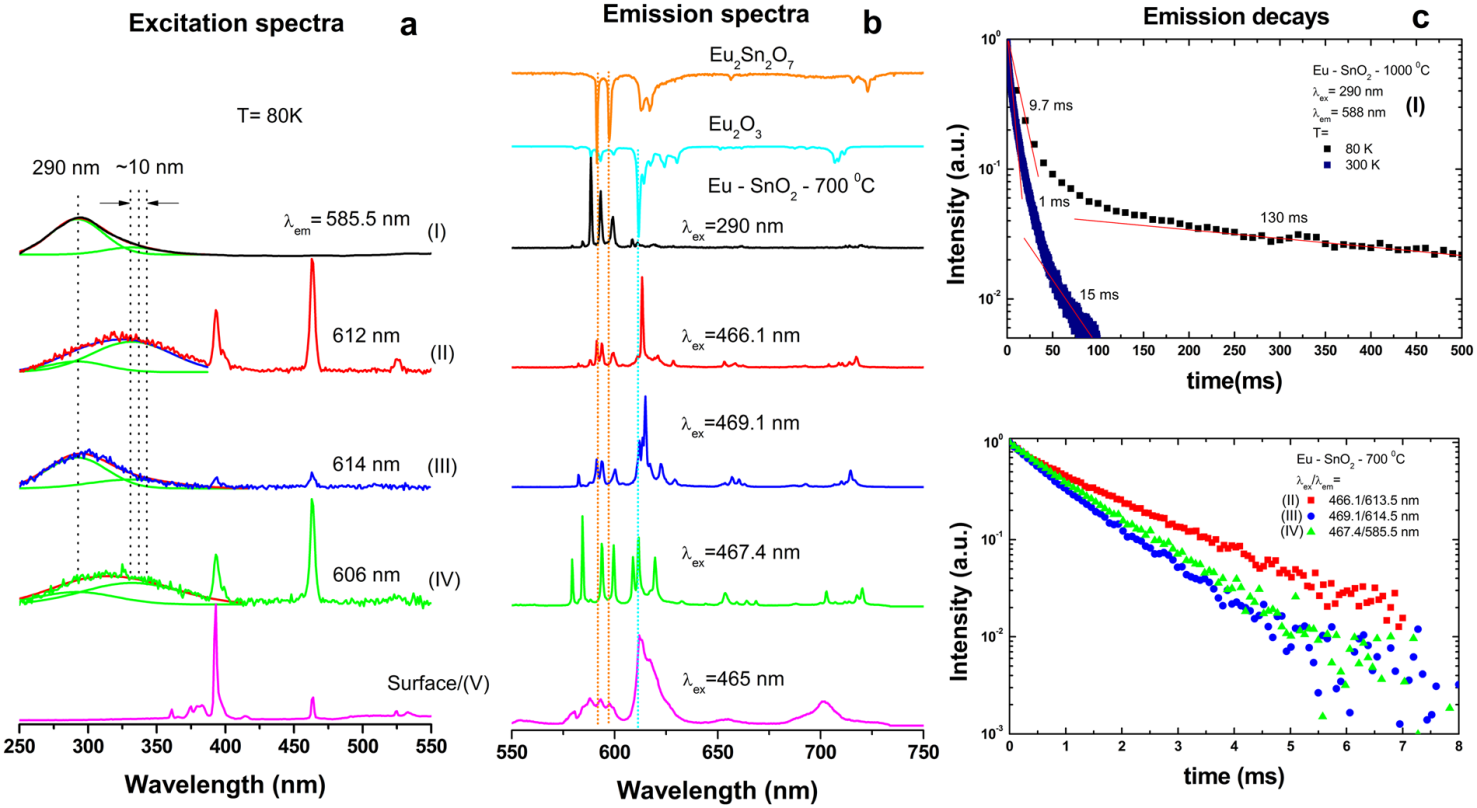
Summary of the emission properties of Eu-SnO_2_ calcined at 700 °C/1000 °C and measured at 80 and 300 K. Characteristic excitation spectra (**a**), emission spectra (**b**) and emission decays (**c**) of Eu centers labelled as **I**–**V centers** (see text). Blue and orange spectra in (**b**) relate to cubic Eu_2_O_3_ and Eu_2_Sn_2_O_7_ pyrochlore, respectively. The guidelines in (**b**) evidence the distinctness of the emission spectra corresponding to** II–IV** centers.

By using a simple deconvolution procedure of the emission spectra^64^ backed also by the analysis of the excitation spectra and emission decays, at least five Sn centers, labelled as **I** to **V** were clearly differentiated (Fig. 5). The emission of center labelled as **I** is exclusively excited *via* SnO_2_ host as shown by the excitation spectrum measured around 588 nm (band at 290 nm). Its shape is characterized by relative strong three ^5^D_0_–^7^F_1_ lines around 590 nm being readily assigned to Eu substituting for the lattice Sn sites (with inversion C_2h_ local symmetry) in line with previous reports^24^. In SnO_2_, the C_2h_ inversion symmetry around Sn cation is shaped by the location of each of the two Sn in the unit cell of SnO_2_ amidst six oxygen atoms which approximately from the corners of a regular octahedron^65^ (Fig. 4). For Eu substituting for Sn with no defect nearby, the emission should be dominated by the allowed MD emission as the electric dipole (ED) transitions are forbidden in the inversion symmetry.

In opposition to this **center I**, a much broader emission, excited exclusively by the f – f absorptions of Eu is observed and assigned to surface type Eu center, labelled as **center V**.

Besides the substitutional isolated C_2h_ (**I**) and surface centers (**V**), at least three more Eu centers, labelled as **II–IV** (or non-C_2h_ centers) could be differentiated for the first time (Fig. 5). The characteristic excitation spectra measured around their emissions around 606–612 nm, show a superposition of broad UV absorption bands around 300–320 nm along with narrow f-f absorptions of Eu. In the excitation spectra, the presence of O^2−^Eu^3+^ charge – transfer band (typically observed between 250–270 nm^66^) may be also masked on the high-energy side of broad UV absorption of SnO_2_ host. There is also a non - negligible interference from the 290 nm band characteristic of C_2h_ center (**I**) due to incomplete spectral separation. The average lifetimes of non - C_2h_ centers (1 ÷ 1.5 ms) are longer than that corresponding to surface center, **V** (0.7 ms), which is an expected result. The surface activators have unsaturated low symmetry coordinations being also subjected to efficient non-radiative energy transfer to surface defects, mainly OH groups still present at 700 and even 1000 °C (Figure S4b). Though the emission lifetime of C_2h_ Eu is expected to be large due to its forbidden MD nature, it exceeds significantly the values of few ms measured for other hosts with inversion symmetry, such as Y_2_O_3_ (S_6_/C_3i_)^47^ or CeO_2_ (O_h_)^48^.

Indeed, as shown in Fig. 5, the emission decay of C_2h_ center displays an exceptional long decay time of 130 ms as estimated from the tail fitting at 80 K. Such a remarkable long-lived emission has recently been assimilated to an atypical persistent emission process that is thermally activated below (or above) 220 K *via* the co-existence of uniform and exponential distributions in trap depths^27^.

To check on the possibility that part of **II**–**IV** emissions relates to impurity phases such as Eu_2_O_3_ or Eu stannate oxide (Eu_2_Sn_2_O_7_), likely induced by the reportedly low solubility limit of Ln in tin oxide lattice, (below 1%), we compared the luminescence of calcined (700/1000 °C) Eu_2_O_3_ and Eu-SnO_2_ measured in identical conditions. There is obviously no resemblance between this emission (included as cyan and orange spectra in Fig. 5) and those of the **II**–**IV** centers. Next, the absence in the Raman spectra of the strongest characteristic phonon mode of stannate pyrochlore (around 510 cm^−1^ ^67^) along with the strongly dissimilar emission exhibited by Eu_2_Sn_2_O_7_^68–70^ grown by us using a sol-gel method^71^ (also included in Fig. 5 as orange spectrum) to Eu centers **II**–**IV** dismiss the occurrence of this phase in our samples.

Further, we asked whether these Eu **II**–**IV** centers were of intrinsic nature or resulted from the procedure of preparation or interaction with specific impurities. Comparison with an additional Eu-SnO_2_ sample grown by a simple sol-gel method give identical emission shapes to those obtained for samples grown by rapid hydrothermal approach. Besides, according to our extensive literature survey on the luminescence of Eu-SnO_2_ published over the last two decades, part of the emission related to centers **II**–**IV** can be observed as strongly non-separated, overlapped emission, though discernable in Eu-SnO_2_ grown by sol-gel^30, 31, 34, 72^ glass-ceramic waveguides^32^, hydrolysis^73^, impregnation and decomposition cycle method^63^, etc. Taken together, the emission shapes and narrowness (few cm^−1^, comparable to that of C_2h_ center) suggest that centers **II–IV** are intrinsic to SnO_2_, resulting from the association of substitutional Eu with nearby bulk defects, most probably oxygen vacancy. Such defects arise from the charge-imbalance on substitution of tetravalent Sn by the trivalent Eu which in the Kroger-Vink^74^ notation is written as:

1 $${{\rm{O}}}_{0}^{x}\to \frac{1}{2}{{\rm{O}}}_{2(g)}+{V}_{{\rm{O}}}^{00}+2{e}^{-}$$

2 $$2{{\rm{Eu}}}^{3+}+2{{\rm{Sn}}}_{{\rm{Sn}}}^{x}+{{\rm{O}}}_{{\rm{O}}}^{x}\to 2{{\rm{Eu}}}_{{\rm{Sn}}}+{V}_{{\rm{O}}}^{00}+{{\rm{Sn}}}_{{\rm{surface}}}.$$

In a further set of experiments, we have surface doped SnO_2_ with Eu by impregnation of pre - calcined pure SnO_2_ at 400 and 1000 °C followed by calcination at 400 and 1000 °C. The XRD patterns, Raman spectra and DR-UV/Vis as well as particle size, lattice constants and estimated band-gap for these additional samples are summarized in Figs 1 and 2, Figures S5 and S6 and Table 2. Despite weak incorporation expected for the pre-calcination-impregnation-calcination procedures, the impregnated samples do show some emission of the substitutional isolated C_2h_ center (**I**) (Figure not shown) embedded into a much broader emission like that of the surface **center V**. However, the notable result is that no emission can be associated to any of the defect centers (**II**–**IV**) giving thus a further confirmation that these centers are induced by Eu substituting for lattice Sn in close association with bulk defects.

Although similar emission properties were measured for Eu-SnO_2_ calcined at 700 and 1000 °C, there are some differences in the details. At higher calcination temperature, both weaker emission and appearance of a distinct distorted emission (Figure S7) suggest a migration of the substitutional Eu towards the nanoparticles surface where they may further associate with adsorbed OH defects^75^. Indeed, the DRIFTS spectra in Figure S4a,b confirm that the surface of the particles is terminated by adsorbed OH (bands centered at 3450 and 1640 cm^−1^) that persist even at 1000 °C and their contribution is exceeding that measured for pure SnO_2_.

**Figure 6:**
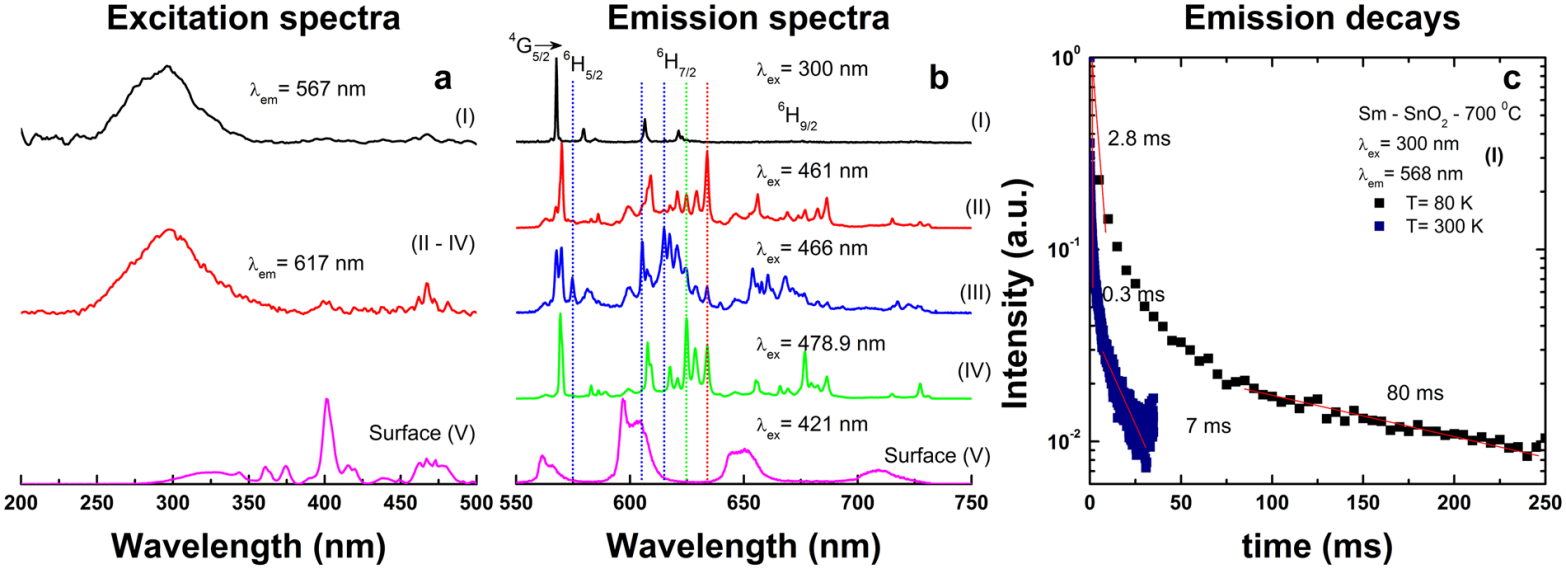
Summary of the emission properties of Sm-SnO_2_ calcined at 700 °C/1000 °C and measured at 80 and 300 K. Characteristic excitation spectra (**a**), emission spectra (**b**) and decays (**c**) of Sm centers labelled as **I–IV** centers (see text). The guidelines in (**b**) evidence the distinctness of the emission spectra corresponding to **II–IV** centers.

The distribution found above for Eu is confirmed by a similar analysis applied to Sm. Compared to Eu, Sm was less frequently investigated as luminescent dopant for SnO_2_^48, 66, 67^. Luminescence studies revealed an effective sensitization of Sm luminescence *via* host absorption^67^ similar to Eu case and also some dependency of the emission shape on the excitation wavelength. Figure 6 summarizes the analysis of luminescence properties of Sm–SnO_2_ calcined at 700/1000 °C that gives similar results for the employed methods (rapid hydrothermal and sol-gel). Under above bandgap excitation (280 nm) the emission of Sm (labelled as **center I**) displays narrow (~0.7 nm) and relative strong ^4^G_5/2_–^6^H_5/2_ emission around 568 nm which is mostly of MD nature ^76^. A weak intensity is observed for the ^4^G_5/2_–^6^H_7/2_ transition at 606 and 621 nm which is of mixed magnetic and electric dipole nature^76^ while no emission could be detected around 670 nm corresponding to the ^4^G_5/2_–^6^H_7/2_ transition which is mostly of electric dipolar nature (ED). In all, the emission shape is consistent with the substitution of Sn by Sm in the inversion C_2h_ symmetry sites with no association with a nearby defect. Our results provide a first evidence of Sm emission assignable to the substitutional isolated C_2h_ center (equivalent to **center I** of Eu). The emission shape is quite similar to that of Sm doped into other hosts with inversion symmetry sites, such as Y_2_O_3_ (S_6_/C_3i_)^76^ and CeO_2_ (O_h_)^48^ recently reported by some of us. In addition, similar to Eu **center I** ^27^ (Fig. 5), the Sm **center I** displays also a remarkable long lived emission decaying on hundreds of ms scale with an estimated decay time from the tail fitting of 80 ms. It appears thus that that the long-lived emission is not Eu specific, given that it belongs to Ln substituting for Sn in the C_2h_ sites (substitutional isolated center). It was previously suggested^27^ that the persistent luminescence of Eu in SnO_2_ may be associated with the co-existence of uniform and exponential distributions in trap depths. It should be noted that most of the persistent luminescence displayed by the lanthanide based phosphors is associated with ED and not to MD transitions^27, 66^.

In addition to the emission assignable to **center I**, we could separate by use of a similar procedure used for Eu- SnO_2_, at least three additional centers characterized by narrow (~0.7 nm) and shorter-lived emission (average lifetime around 1 ms) and labelled as centers II–**IV** in Fig. 6. The separation of the excitation spectra and decays of **centers II**–**IV** of Sm was however more difficult than in the case of Eu, therefore we have included only one excitation spectrum in Fig. 6. The average lifetimes of Sm centers **II–IV** is roughly estimated between 0.5 and 1ms. A broad and strongly distorted emission was observed and readily assigned to Sm surface center (corresponding **center V** of Eu). In addition to **centers I** and **V**, corresponding to isolated substitutional and surface centers of Eu and Sm, respectively, we can tentatively suggest a common nature also for **centers IV** of Eu and Sm. Both **centers IV** of Eu and Sm display a relative strong intensity of MD transition (^5^D_0_–^7^F_1_ for Eu and ^4^G_5/2_–^6^H_5/2_ for Sm) suggesting that the coordination around Eu and Sm centers is slightly deviated from the inversion C_2h_ symmetry. Taken together, the luminescence properties of Eu and Sm in SnO_2_ are consistent with a common distribution, irrespective of the synthesis method, that includes substitutional isolated, associates with defects and surface centers.

We have also investigated the luminescence of Pr, Tb and Dy-SnO_2_ samples in the as-synthetized state and after calcination at 400 and 700 °C. Selected XRD patterns and DR-UV/Vis spectra as well as luminescence spectra of Pr, Tb and Dy-SnO_2_ are gathered in Figures S8 and S9, respectively. To the best of our knowledge, there is no literature report on Pr emission in SnO_2_, while for Tb^44^ or Dy^45, 46^ doped SnO_2_ the emissions appear to be related to surface location rather than substitutional doping. As a general observation, we note that the intensities of the Pr, Tb and Dy related emissions were poor, precluding advanced investigation. The emissions relate possibly to ^1^D_2_ level of Pr and definitely to ^5^D_4_ or^4^F_9/2_ levels of Tb or Dy. To decide whether the observed emission may relate to isolated substitutional doping, we included, in Figure S9 the emission spectra of Tb and Dy in the inversion sites of Y_2_O_3_ (S_6_/C_3i_) and CeO_2_ (O_h_) reported recently by some of us^47, 48^. It can be observed that the emission shapes of Tb and Dy depart strongly from those expected for an inversion symmetry. Besides, the emissions are broad (~13 nm for Dy and ~9 nm for Tb) and short-lived (up to few hundreds of microsec). We therefore concluded that, that in contrast to Eu and Sm, Pr, Tb and Dy in SnO_2_ cannot substitute for Sn in the inversion lattice sites, preferring instead highly distorted environments, likely connected with the SnO_2_ surface.

### Particle size effect on the local structure

**Figure 7:**
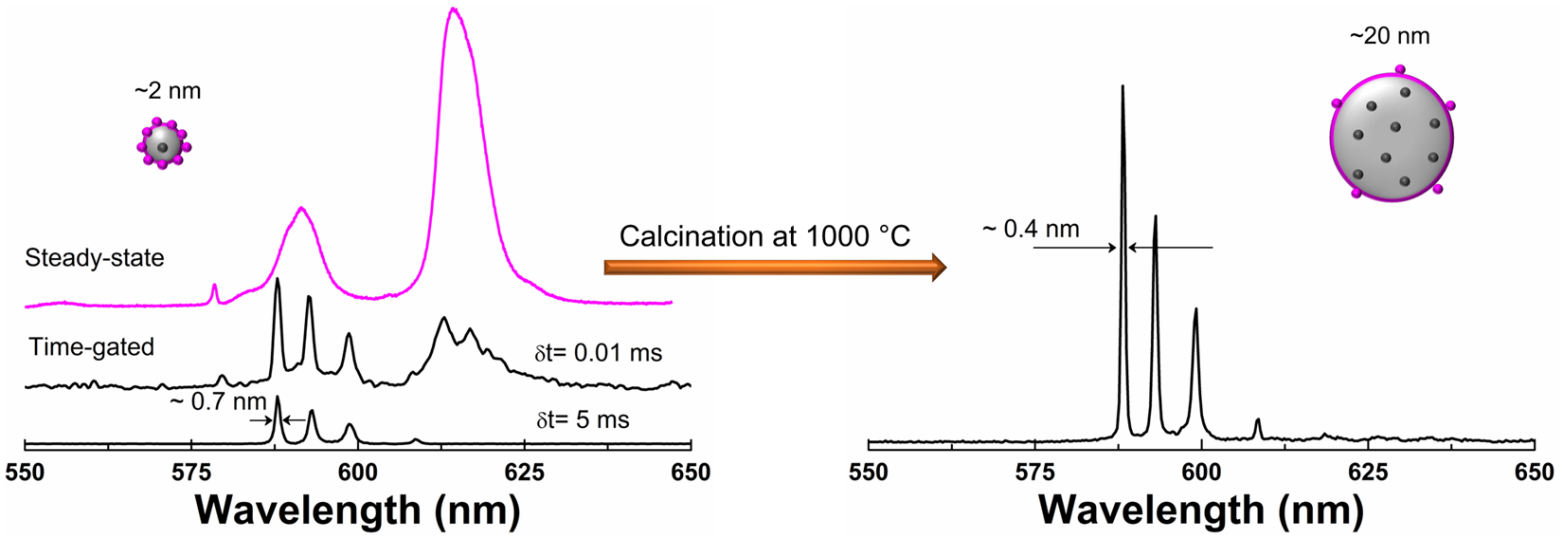
Schematic representation of the **s**imilar local structures around Eu in SnO_2_ in the 2 and 20 nm sized SnO_2_ suggested by time-gated luminescence approach (δt notation means delay after the laser pulse).

It is well established that, as the particle size decreases, the increasing number of surface and interface atoms generates stress/strain and concomitant structural perturbations^28^. The comparison of luminescence properties of small (mean particle size comparable to the bulk exciton Bohr radius) and large sized Eu-SnO_2_ nanoparticles led to the conclusion that the Eu environment is strongly distorted in the small SnO_2_ while it is crystalline in the larger nanoparticles^29^. We re- asses here the particle size effect on the local structure selecting Eu-SnO_2_ particle sizes around 2 nm (as-synthetized) and 20 nm (calcined at 1000 °C). Figure 7 compares the steady-state emission spectra of the two Eu- SnO_2_ nanoparticles (excited above bandgap around 300 nm) that evidence a strongly distorted emission for the smaller sized nanoparticles (magenta spectrum). This may be used as a spectroscopic evidence that the local environment gets more distorted with decreasing particle size. However, using the time gated (delayed) mode, a narrow, long-lived type emission with orders of magnitude less intensity than the broad emission could be extracted with the 2 nm sized particles. Except for the slightly different widths, the delayed emission spectrum measured with the 2 nm particle is quite similar to that measured with the 20 nm particle. Both emissions correspond to the substitutional isolated Eu center (**center I**) with similar values of emission peaks (588.24, 593.04 and 599.08 nm) and intensity ratio (1/0.76/0.59) suggesting similar atomic scale environments of the inner lattice Sn, irrespective of the particle size. The reported distortions likely arises from the generic “surface effect” which is responsible for a broad and short-lived luminescence^48^ observed with wide range of doped nanoparticles, irrespective of host and luminescent activator.

We also highlight here that the advantage of using time-gated luminescence as a local technique over long-range XRD and medium range Raman techniques in detecting weak crystallinity features is obvious. In the Figs 1b and 2b only broad, non-crystalline patterns and surface phonon modes are observed for the as -synthetized Eu- SnO_2_ making difficult to assess the substitutional doping effect.

### Surface *versus* bulk doping in the photocatalytic behavior

**Figure 8:**
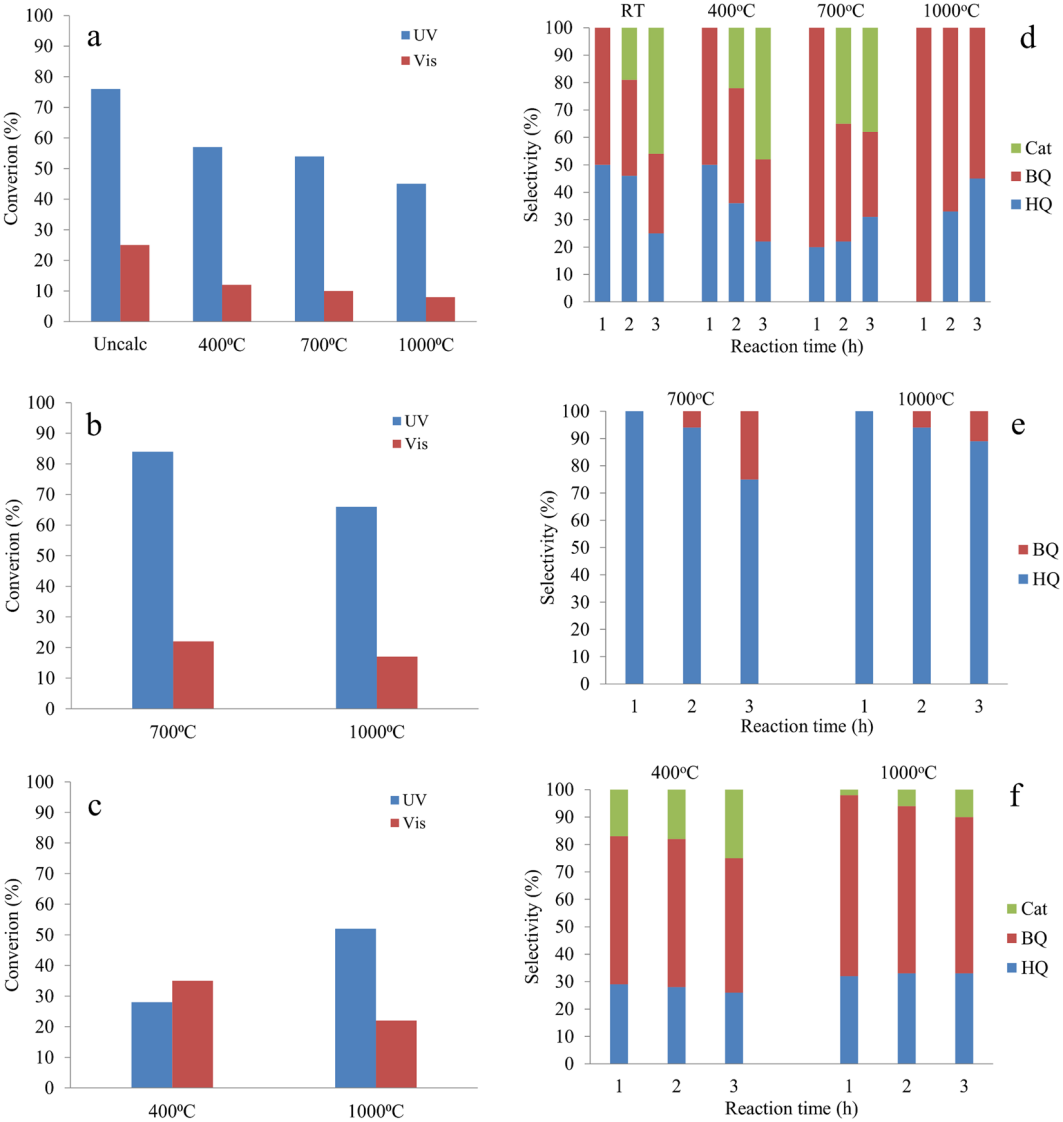
Conversion of phenol under 3 hours of UV and Vis irradiation using (**a**) SnO_2_, (**b**) Eu-SnO_2_ and (**c**) Eu-SnO_2_ (impregnated) and selectivity to Hydroquinone (HQ), 1,4 Benzoquinone (BQ) and Catechol (Cat) under UV irradiation using (**d**) SnO_2_, (**e**) Eu-SnO_2_ (conventional doping) and (**f**) Eu- SnO_2_ (impregnated) function of calcination temperature.

Recent literature shows that the Ln dopants increase the photocatalytic activity of SnO_2_ in the photo-oxidation of phenol under UV or solar simulated light^5^. Our photocatalytic measurements used here as an additional probe shows that pure, doped and impregnated Eu-SnO_2_ exhibit catalytic activity in the oxidation of phenol (Fig. 8a–c, Figure S11) irrespective of the calcination temperature and UV versus Vis irradiation range (see also Figure S1 for the spectral excitation profiles). The conversion decreased with the increase of the temperature in opposition to reports on non-structured catalysts^15^ being, however in line with textural analysis (surface and pore blockage by carbonaceous products and collapse of the structure by calcination (Table 1) and DR-UV-Vis measurements (Figure S5). Decrease of the conversion efficiency with calcination temperature (reflected in the decrease of the surface area, increase of the crystallite sizes (Table 1) and slight increase in the band gap (Table 2)) are due to modification of defects population in both pure and Eu doped/impregnated SnO_2_. The advantage of the materials structuration is demonstrated by the higher catalytic conversions of doped Eu-SnO_2_ (Figure S11b) and the smaller photocatalytic conversions on the impregnated Eu-SnO_2_ (Figure S11c) compared to pure SnO_2_. Although Eu presence does not affect strongly the band gap (Table 2), its dispersion in the bulk is generating a higher photoactivity compared to pure and impregnated samples. The luminescence investigations evidenced a migration of the lattice Eu towards the surface with increase of calcination temperature from 700 to 1000 °C while the impregnated samples exhibit mainly the surface defects (surface **center V**, Fig. 5) that along with a slightly wider band gap is reflected in a lower activity compared with the doped samples. Although photocatalysis is a surface process, the photons can penetrate at a certain depth in the nanoparticle where the presence of bulk defects may be beneficial. The difference of only 0.2 eV in the band-gap is not enough to explain the increased conversion with doped Eu-SnO_2_ compared to pure SnO_2_. Moreover, the impregnated Eu-SnO_2_ exhibits a higher band-gap than pure SnO_2_ or doped Eu-SnO_2_. On the other side, the activity of the impregnated samples increases with the increase in the temperature (pre-calcination and subsequent calcination) (Figure S11c), that is in agreement with previous reports^15^. The activity under Vis irradiation (allowed by the spectral profile illustrated in Figure S10) exceeds by almost 300 and 50% those of doped and pure SnO_2_ for the same (pre)calcination temperature of 400 °C. At 1000 °C, the Vis activity is comparatively weaker than at 400 °C which arises likely for the compromise of larger band gap (3.2 compared to 2.6 eV, Table 2) and reduction of bulk defects over surface ones (as Eu incorporation into the lattice is hindered by the higher pre-calcination temperature).

**Figure 9:**
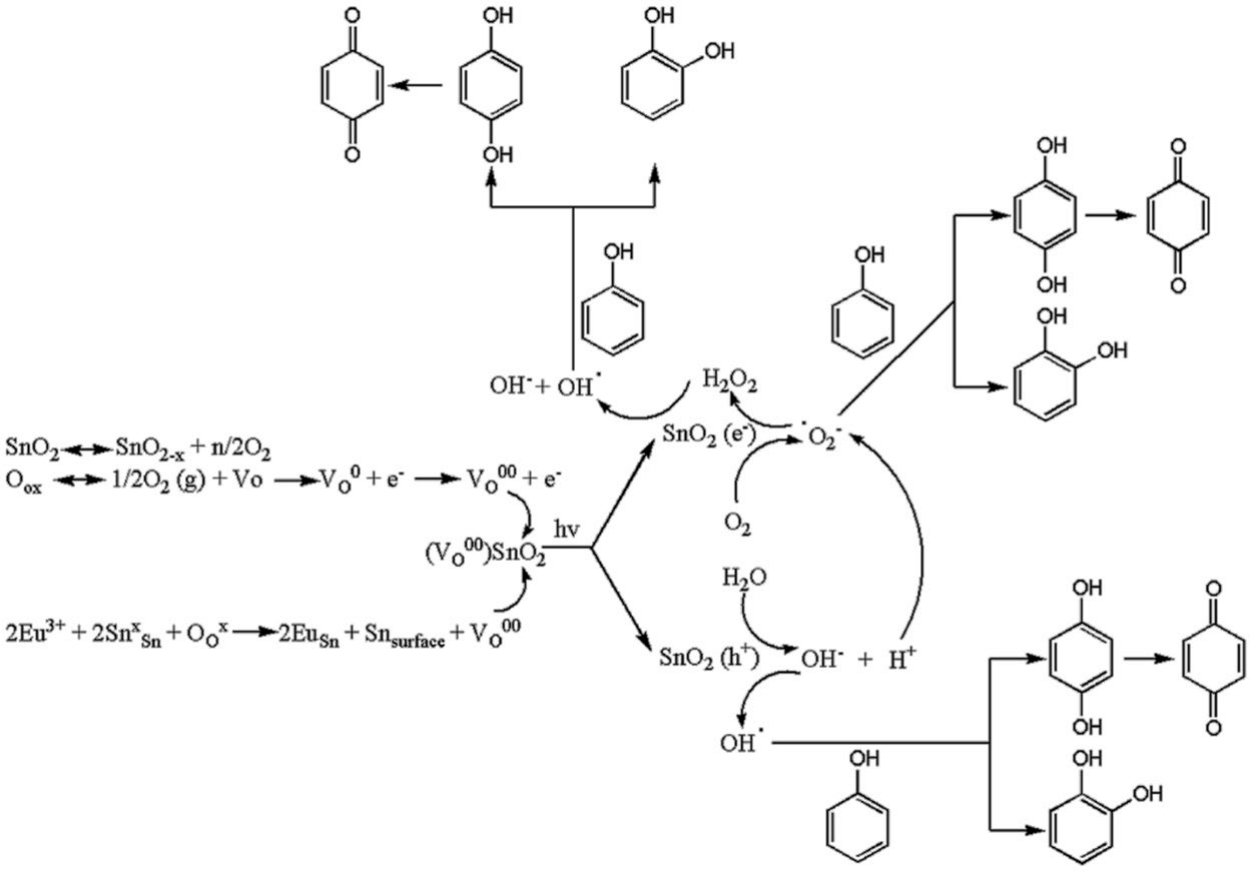
Reaction pathways for the photo-oxidation of phenol with Eu-SnO_2_.

Figure 8d–f shows the evolution of the selectivity towards hydroquinone (HQ), catechol (Cat) and 1, 4-benzoquinone (BQ) intermediates^77, 78^ Under UV irradiation, the reaction with pure SnO_2_ advanced producing both BQ and Cat (Fig. 8d) with no Cat being produced after one hour of irradiation. In concordance to the above comments concerning the decrease in the population of the defects and changes in the band gap, the sample calcined at 400 °C afforded HQ and BQ in the first hour of irradiation, Cat being produced only after two hours, the sample calcined at 700 °C, after one hour afforded mainly HQ, and calcination at 1000 °C led to HQ as major product (100% selectivity after one hour), Cat not being produced. In all the cases, the plots suggest a consecutive/parallel oxidation process (Fig. 9). Doping SnO_2_ with Eu (Fig. 8e) changed the selectivity predominantly in the favor of HQ (see samples calcined at 700 and 1000 °C, respectively) following the same mechanism and preserving the effect of the calcination. However, the parallel reaction is slower in this case, with no Cat being produced. For the impregnated Eu-SnO_2_, a small amount of Cat is also detected (Fig. 8f) which is lower for the sample treated at 1000 °C than 400 °C following the same trend as pure SnO_2_.

For both pure and Eu-SnO_2_ (doped or impregnated) samples, under Vis irradiation, the reaction stopped at hydroquinone (HQ) with complete selectivity (not shown). As the results showed, the presence of surface and bulk defects is beneficial for the photocatalytic performance of the samples. The neutral oxygen vacancies (Vo) in the structure of SnO_2_ that are produced by elimination of water during the Sn(OH)_4_ – SnO_2_ transition^15^. Although the insertion of Eu is supposed to enhance the population of the defects, except for the samples calcined at high temperatures, the band-gap remained basically unchanged. According to the equations included in Fig. 9, phenol can be oxidized by the O_2_^−^ species and OH radicals generated on the defected sites under the light irradiation. In all, the results described above suggest that the correlation of luminescence and photocatalytic properties, being both defects -related, can be used as an additional tool for improving the identification of bulk over surface defects in trivalent metal doped SnO_2_. Generally, the substitution of metal cations in oxide lattice by metal ions with different valence states disrupts the chemical bonds in the host oxide generating superficial active centers represented either by the oxygen atoms near the dopant or the dopant itself^79^. Low valence dopants such as the lanthanide ions, activate the near oxygen atoms, thus the doped host oxide is a better oxidant than the undoped one. The effect of higher valence dopants depends on the nature of the host oxide^79^. For irreducible oxides (e.g. Ta_2_O_5_), the host surface oxygen atoms are less active and the oxidation takes place merely by the surface adsorbed and activated O_2_. When the host oxide is reducible (e.g. TiO_2_, CeO_2_, SnO_2_), the dopant metal will donate electrons to the host cations (for the case of SnO_2_, reducing their valence as Sn4+ → Sn2+). For SnO_2_, in addition to the generation of active sites, the electronic control of energy levels in dopant clusters is involved in the activation of certain reactions by dopants^80^. On the other hand, the particle size of the doped metal oxide becomes smaller than the pure host oxide, as consequence of restriction of the motion of crystallites at the interaction on the boundaries between host and dopant crystallites^81^, as it can be seen in Table 2.

## Conclusions

We have investigated pure and trivalent lanthanide doped SnO_2_ during and at various calcination temperatures up to 1000 °C, by use of a suite of *in situ*/*ex situ* techniques and methods. It is found that, before calcination most Eu resides on nanoparticle surface while mild calcination up to 500 °C lead to a significant reduction of surface effects. Besides, the redistribution of dopants along with induced defects remain limited to the surface layer of 2–6 nm sized particles. At higher calcination temperatures (≥700 °C) a complex distribution as substitutional isolated, associates with bulk defects and surface centers was evidenced for both Eu and Sm. The manifestation of a remarkable long-lived luminescence decaying on the hundreds of ms scale for both ions suggested that such phenomenon is not lanthanide specific, being tentatively associated to interaction between traps and the substitutional isolated Ln center. In contrast to Eu and Sm, Pr, Tb and Dy lanthanide dopants distributed mostly on the surface of SnO_2_ nanoparticles as judged by their characteristic emission. As both luminescence and photocatalytic properties are defects -related, their correlation proved effective in distinguishing the surface over bulk defects and confirming the temperature induced migration of dopants from the lattice sites towards the surface.

## Materials and Methods

### Synthesis

The synthesis of SnO_2_ nanoparticles was carried out by a rapid hydrothermal and sol-gel methods. In the first approach, a quick introduction of one gram of solid tin tetraisopropoxide isopropanol solvate (Multivalent Co UK) into 50 ml of boiling Milli-Q water with subsequent refluxing of the obtained milky dispersion for 30 min is used^16^. The mixture was then left for precipitation overnight, the supernatant was removed by decantation and the solid residue was dried at room temperature in air overnight. The Ln (Sm, Eu) doped SnO_2_ nanopowders were produced by the same procedure using instead of pure water a solution of stoichiometric amount of Ln(NO_3_)_2_ in 50 ml of water. Ln (1 wt%) was also deposed on pre-calcined SnO_2_ (at 400 and 1000 °C, respectively) *via* the wetness impregnation with an aqueous solution of Ln(NO_3_)_3_∙6H_2_O (Alfa Aesar) followed by drying overnight at room temperature and calcination at 400 and 1000 °C, respectively. In the sol-gel method, Ln(Eu, Sm, Pr, Tb and Dy, 1 wt%) doped SnO_2_ nanopowders were prepared by dissolving of 2 g stannous chloride dihydrate (SnCl_2_.2H2O) and the appropriate amount of dopant Ln(NO_3_)_3_∙6H_2_O in 100 ml distilled water. After complete dissolution, ammonia solution was added to the above solution by drop wise under stirring. The suspensions were left overnight for ageing, centrifuged and then dried at room temperature for 24 hours and 60 °C for 4 hours. The samples were calcined in air at the desired temperature (400, 700 and 1000 °C) with a rate of 10 °C/min.

### Characterization

Microbeam X-ray fluorescence (micro-XRF) spectrometry was performed on a custom-made instrument with an X-ray tube: Oxford Instruments, Apogee 5011, Mo target, focus spot ∼40 µm, max. high voltage −50 kV, max current −1 mA, Amptek X-123 complete X-Ray spectrometer with Si-PIN detector. The key element of the micro-XRF instrument is an X-ray policapillary minilens (IfG-Institute for Scientific Instruments) which provides a focal spot size on the sample of 15–20 µm. X-Ray Diffraction (XRD) measurements were recorded on a Shimadzu XRD-7000 diffractometer using Cu Kα radiation (λ = 1.5418 Å, 40 kV, 40 mA) at a scanning speed of 2 degrees min^−1^ in the 10–90 degrees 2θ range. *In-situ* experiments were carried out in a cell accessory in the 50–1000 °C temperature range. The heating rate was 10 °C/min and the samples were kept for 10 min at the set temperature before collecting the diffractograms. The Raman spectra were acquired in the extended spectral region from 150 to 4000 cm^−1^. Raman analysis was carried out with a Horiba JobinYvon - Labram HR UV-Visible-NIR Raman Microscope Spectrometer, at 514 nm. *In situ* Raman spectra were recorded using the same apparatus equipped with an *in-situ* cell, until 600 °C. The heating rate was 10 °C/min and the sample was kept for 10 min at each temperature before collecting the spectra. Diffuse reflectance optical (DR-UV-Vis) spectra were recorded at room temperature on a Analytik Jena Specord 250 spectrophotometer with an integrating sphere for reflectance measurements and MgO as the reflectance standard. DR-UV-Vis spectra of the materials were recorded in reflectance units and were transformed in Kubelka–Munk remission function F(R). The estimation of the band-gap energy was made using the E = hc/λ (eV) formula, where h is the Planck constant (4.135 • 10^−15^ eVs), c is the speed of the light (3 • 10^8^ ms^−1^), and λ the absorption threshold value for each sample determined from the spectra. The value of E_g_ (*ie* the band gap of the semiconductor) was derived from the spectra by plotting (F(R) • hν)^2^ against hν^18^. Diffuse Reflectance Fourier Transform Infrared (DRIFT) spectra were measured with a Thermo Electron Nicolet 4700 FTIR spectrometer with a Smart Accessory for diffuse reflectance measurements. The IR spectra were scanned in the region of 4000–400 cm^−1^ at the resolution of 4 cm^−1^. The final spectra are an accumulation of 400 scans. Transmission electron microscopy (TEM) investigations were made using a Tecnai G^2^ T20 microscope (FEI^TM^, Holland) with an accelerating voltage of 200 kV. Scanning electron microscopy (SEM) studies were carried out with Hitachi TM 1000 tabletop environmental microscope equipped with μ-DeX EDS detector system.

### Luminescence measurements

The photoluminescence (PL) measurements were carried out using a Fluoromax 4 spectrofluorometer (Horiba) operated in both the fluorescence and the phosphorescence mode. The repetition rate of the xenon flash lamp was 25 Hz and 1 Hz for persistent luminescence decay, the integration window varied between 0.1 and 0.5 s, the delay after flash varied between 0.03 and 500 ms, and up to 30 flashes were accumulated per data point. For excitation spectra, the slits were varied from 5 to 29 nm while in emission the slits were varied from 1 nm to 3 nm. The PL decays were measured by using the “decay by delay” feature of the phosphorescence mode. The average decay time was calculated as integrated area of normalized decay. Time resolved emission spectra were recorded using a wavelength tunable (from 210 to 2300 nm) NT340 Series EKSPLA OPO (Optical Parametric Oscillator) operated at 10 Hz as excitation light source. The tunable wavelength laser has a narrow linewidth <4 cm^−1^ with scanning step varying from 0.05 to 0.1 nm. As detection system, an intensified CCD (iCCD) camera (Andor DH720) coupled to a spectrograph (Shamrock 303i, Andor) was used. The time-resolved emission spectra were collected in the spectral range of 450 nm < λ_em_ < 850 nm using the box car technique. The luminescence measurements were recorded at low-temperature (80 K) by use of FL-1013 Liquid Nitrogen (LN) dewar assembly (Horiba) and at 300 K.

### Photocatalytic tests

Photocatalytic tests. In a typical phenol oxidation experiment, 15 mg of photocatalyst were added to a 5 mL aqueous solution of 50-ppm phenol. Two UV lamps (purchased from Vilber Lourmat) centered at 254 nm (VL-340.G 120 W) and 365 nm (VL-340.BL 120 W), respectively, were used as UV light sources for reactions under UV irradiation, and one 150 W Philips Master Colour CDM-T 150 W/830 visible lamp was used as visible light source for reactions under visible light irradiation. 30 μL of solution was collected at 60 min intervals for high performance liquid chromatography (HPCL, Agilent Technologies 1260 Infinity with DAD detector. Column Eurosphere C18, flow rate 1 mL/min, CAN: H_2_O = 40: 60, λ = 274.5 nm, Vinj = 10 µL).

### Data availability statement

The datasets generated during and/or analyzed during the current study are available from the corresponding author (CT) on reasonable request.

## Electronic supplementary material

Supplementary Information Nanoscale insights into doping behavior, particle size and surface effects in trivalent metal doped SnO2
