# Supplementary material for: Nanoscale insights into doping behavior, particle size and surface effects in trivalent metal doped SnO2
Source: Sci Rep. 2017 Aug 29;7:9598. doi: 10.1038/s41598-017-09026-2 (PMC5575111; doi:10.1038/s41598-017-09026-2)
Supplement: Supplementary file 1 — Supplementary Information Nanoscale insights into doping behavior, particle size and surface effects in trivalent metal doped SnO2 [file 41598_2017_9026_MOESM1_ESM.pdf]

Supplementary Information

Nanoscale insights into doping behavior, particle size and surface effects in trivalent metal doped SnO_2

Bogdan Cojocaru,[a] Daniel Avram[b], Vadim Kessler[c], Vasile Parvulescu[a], Gulaim Seisenbaeva[c], Carmen Tiseanu[b*]

[a] *Department of Chemistry, Univ. Bucuresti, B-dul Regina Elisabeta, nr. 4-12, Sector 3, 030018 Bucharest, Romania*

[b] *National Institute for Laser, Plasma and Radiation Physics, P.O. Box MG-36, RO 76900, Bucharest-Magurele, Romania*

[c] *Department of Molecular Sciences, Biocenter, SLU, Box 7015, SE-75007, Uppsala, Sweden*

[*]*Author to whom correspondence should be addressed. Electronic mail:* carmen.tiseanu@inflpr.ro

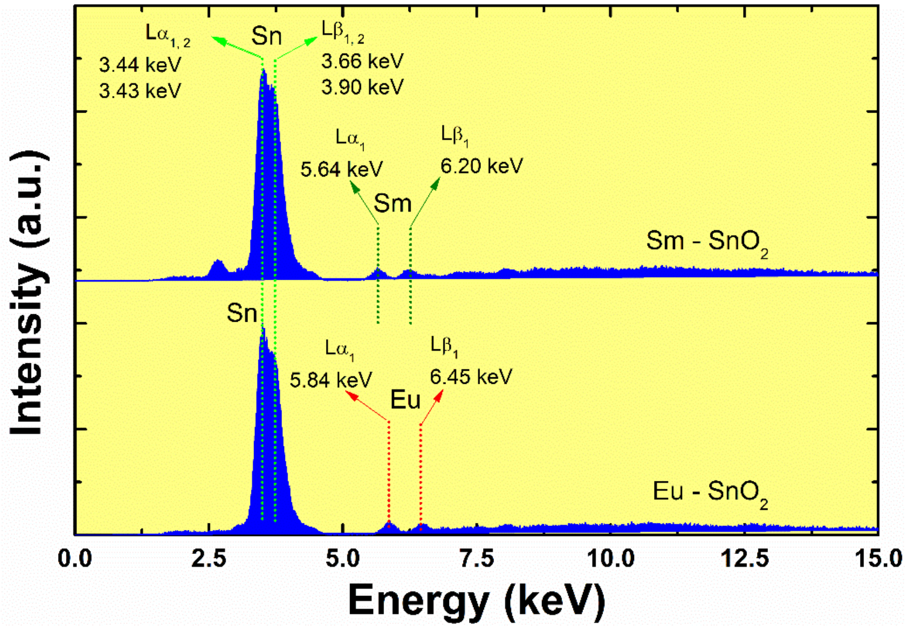

Figure S1. X-ray Fluorescence (XRF) spectra of as-synthetized Eu - SnO_2 and Sm - SnO_2.

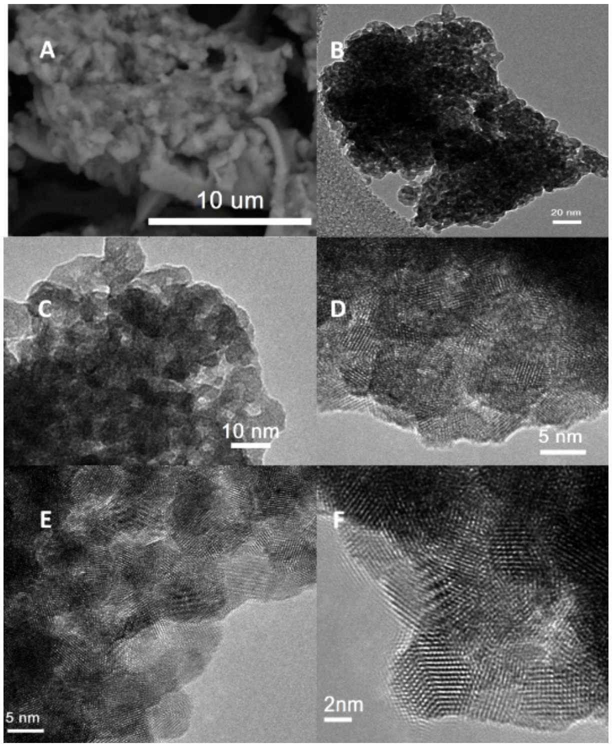

Figure S2. SEM view of the as-synthetized SnO_2 particles (A), TEM images of the same material with increasing magnification (B-D), TEM images of the material heat-treated at 400°C (E,F).

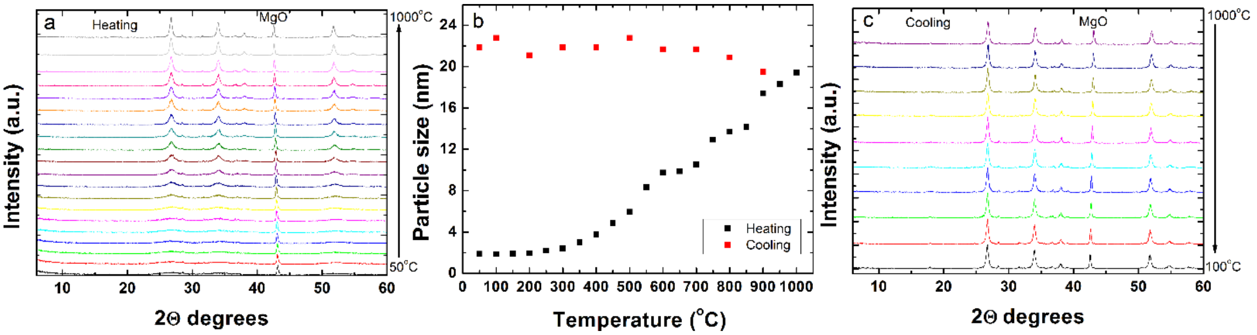

Figure S3. In situ XRD patterns (a,b) and variation of the particle size of Eu – SnO_2 during heating and cooling cycles (c).

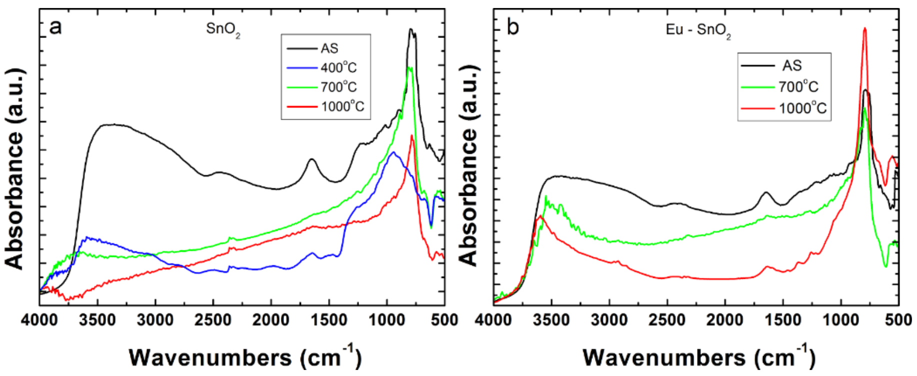

Figure S4. DRIFTs spectra of SnO_2 (a) and Eu – SnO_2 (b).

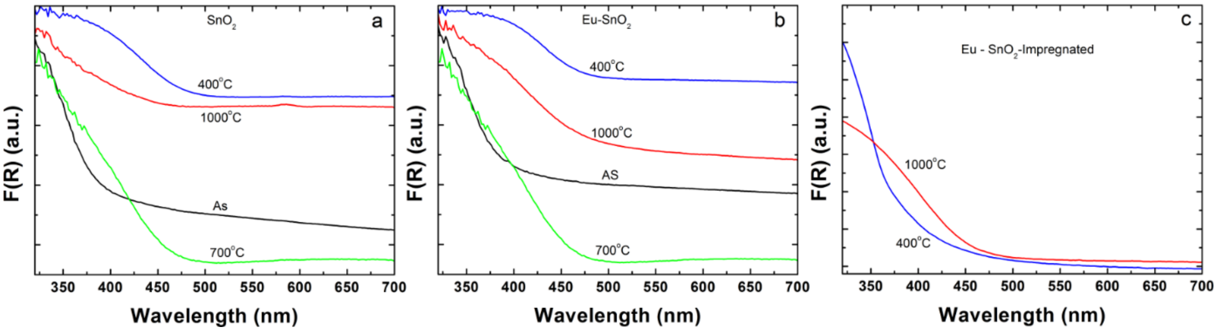

Figure S5. DR – UV/Vis spectra of SnO_2 (a), Eu – SnO_2 (b) and impregnated Eu – SnO_2 (c). The decomposition of the precursors by calcination led to the formation of carbonaceous deposits which correspond to a change in color from white to green - grey below 400 ºC**.**

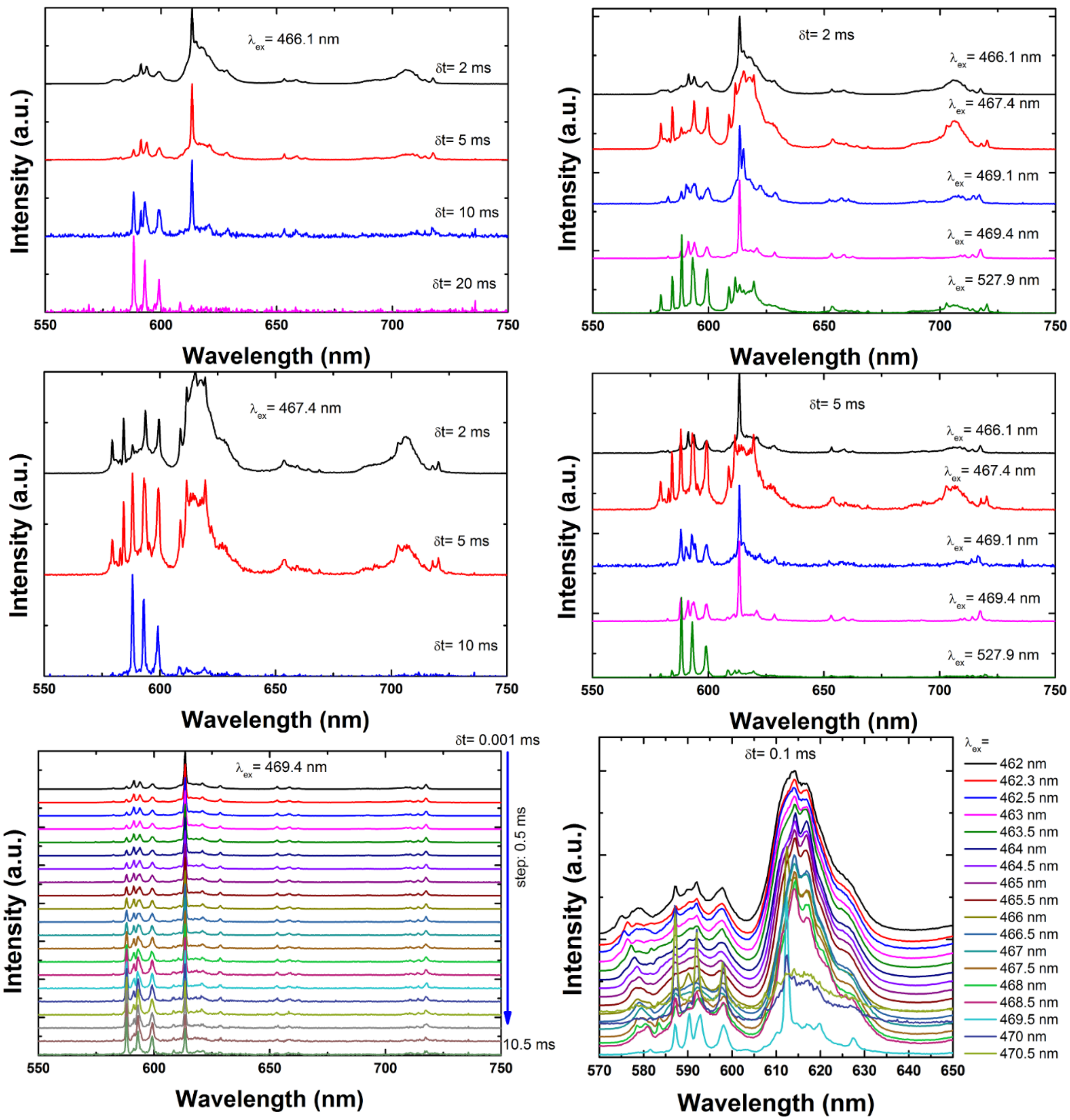

Figure S6. Selected emission spectra used for the spectral separation of the Eu **I – V** centers illustrated in Figure 5.

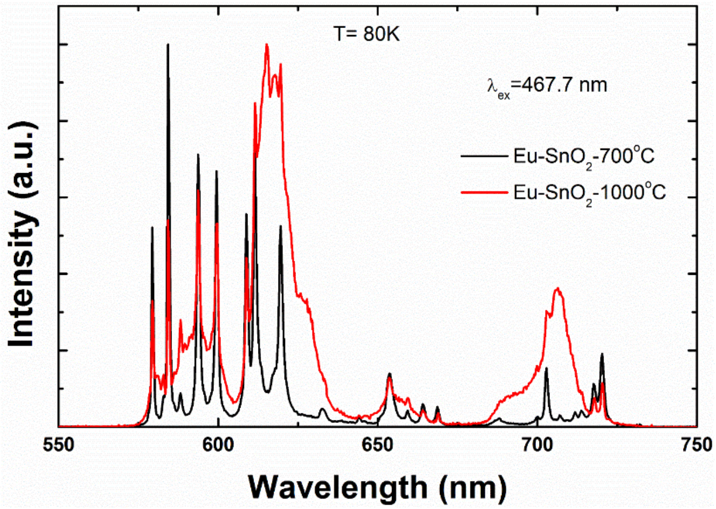

Figure S7. Comparison between the emission spectra of Eu – SnO_2 calcined at 700 and 1000 °C showing an enhanced contribution of Eu-surface defect associate (likely OH, see text) in sample calcined 1000 °C.

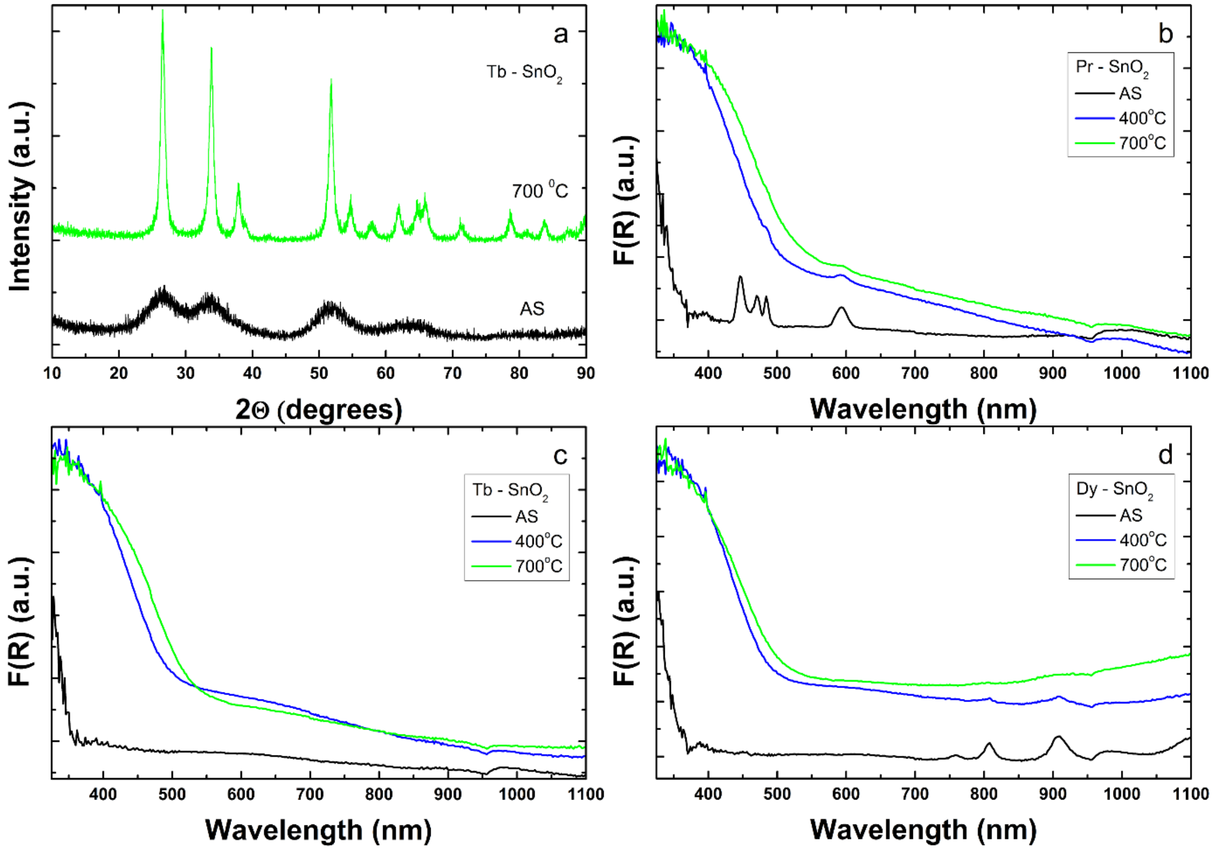

Figure S8. Selected XRD patterns (a) and DR-UV/Vis spectra (b) of Pr, Tb and Dy-SnO_2. f – f absorption transitions can be readily observed for Pr and Dy in the DR-UV/Vis spectra (b, d).

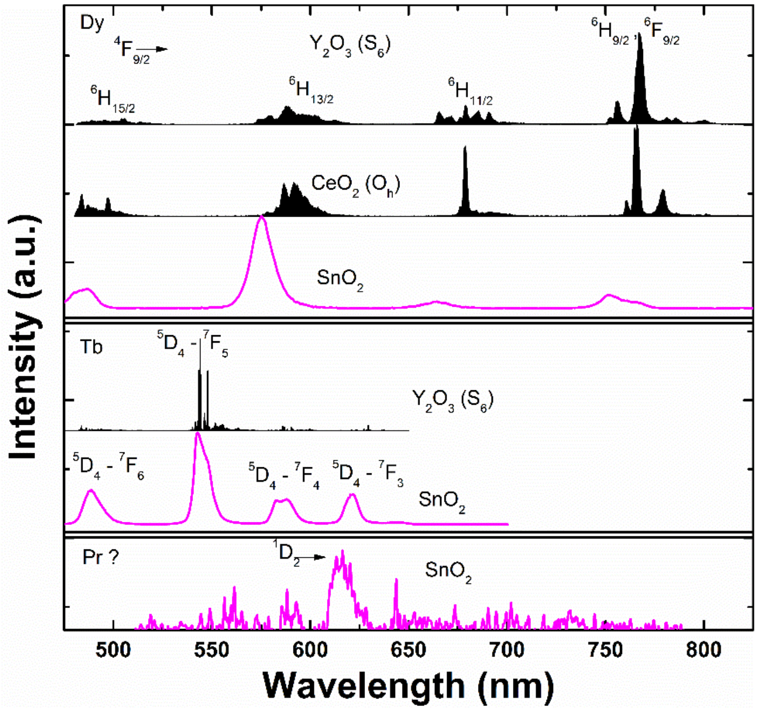

Figure S9. Luminescence spectra (c) of Tb and Dy-SnO_2. For comparison purpose, included are the emission spectra of Dy and Tb in the inversion symmetry sites of Y_2O_3[1] and CeO_2[2].

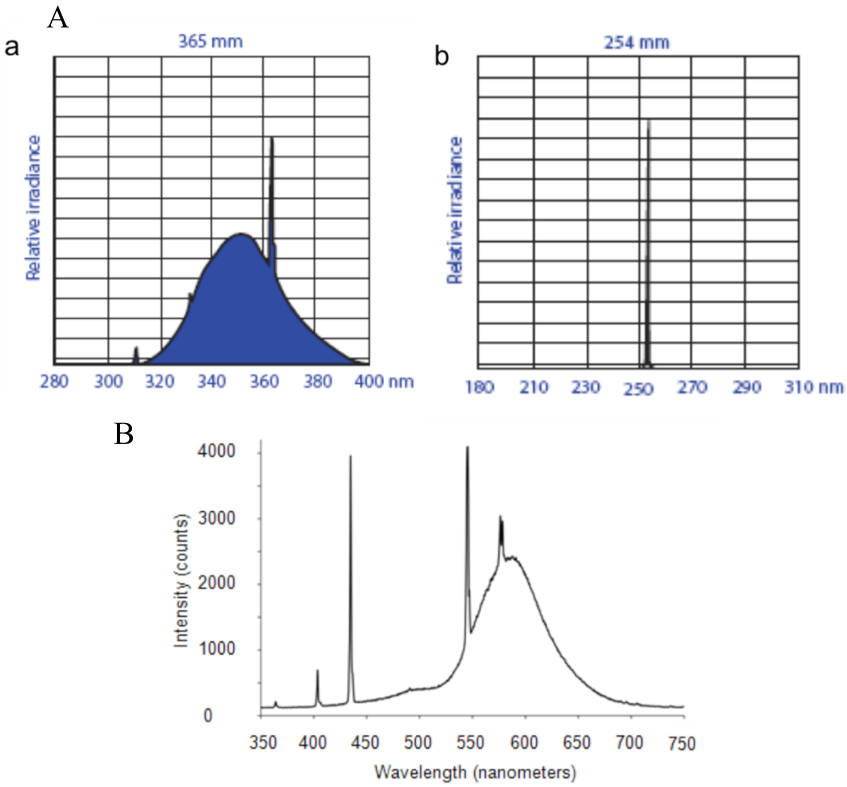

Figure S10. The emission spectra of the lamps: A. The UV irradiation is composed of (a) and (b); B. visible light irradiation.

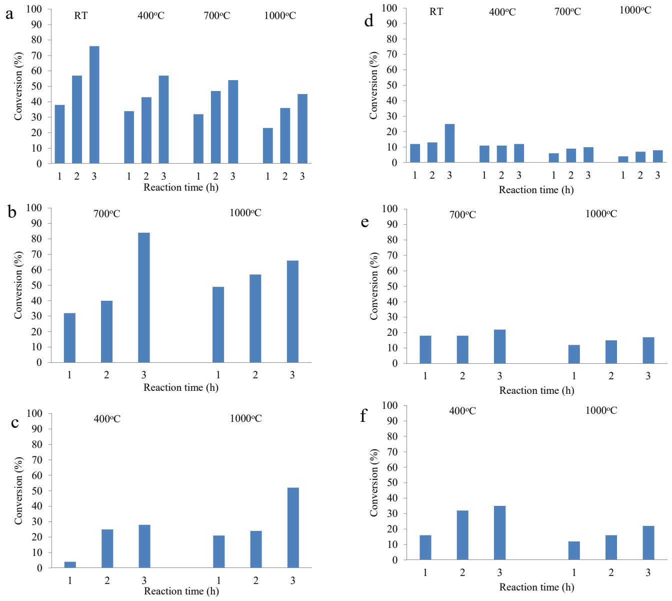

Figure S11. Conversion of phenol under under UV irradiation using a. SnO_2, b. D-Eu - SnO_2 and c. I-Eu - SnO_2 and Vis irradiation using d. SnO_2, e. D-Eu - SnO_2 and f. I-Eu - SnO_2 as function of calcination temperature.

References:

1 Avram, D., Cojocaru, B., Florea, M. & Tiseanu, C. Advances in luminescence of lanthanide doped Y_2O_3: case of S_6 sites. *Optical Materials Express* **6**, 1635-1643, doi:doi:10.1364/OME.6.001635 (2016).

2 Avram, D. *et al.* Toward a Unified Description of Luminescence–Local Structure Correlation in Ln Doped CeO_2 Nanoparticles: Roles of Ln Ionic Radius, Ln Concentration, and Oxygen Vacancies. *The Journal of Physical Chemistry C* **119**, 16303-16313, doi:10.1021/acs.jpcc.5b02240 (2015).
